# Systematic optimization of fermentation conditions for *in vitro* fermentations with fecal inocula

**DOI:** 10.3389/fmicb.2023.1198903

**Published:** 2023-07-24

**Authors:** Jonas Poppe, Sara Vieira-Silva, Jeroen Raes, Kristin Verbeke, Gwen Falony

**Affiliations:** ^1^Translational Research in Gastrointestinal Disorders (TARGID), Department of Chronic Diseases and Metabolism, KU Leuven, Leuven, Belgium; ^2^Institute of Medical Microbiology and Hygiene and Research Center for Immunotherapy (FZI), University Medical Center of the Johannes Gutenberg-University Mainz, Mainz, Germany; ^3^Institute of Molecular Biology (IMB), Mainz, Germany; ^4^Laboratory of Molecular Bacteriology, Department of Microbiology and Immunology, KU Leuven, Leuven, Belgium; ^5^Center for Microbiology, VIB-KU Leuven, Leuven, Belgium; ^6^Leuven Food Science and Nutrition Research Center (LFoRCe), KU Leuven, Leuven, Belgium

**Keywords:** fermentation, *in vitro*, microbiome, optimization, prebiotic

## Abstract

*In vitro* fermentation strategies with fecal inocula are considered cost-effective methods to gain mechanistic insights into fecal microbiota community dynamics. However, all *in vitro* approaches have their limitations due to inherent differences with respect to the *in vivo* situation mimicked, introducing possible biases into the results obtained. Here, we aimed to systematically optimize *in vitro* fermentation conditions to minimize drift from the initial inoculum, limit growth of opportunistic colonizers, and maximize the effect of added fiber products (here pectin) when compared to basal medium fermentations. We evaluated the impact of varying starting cell density and medium nutrient concentration on these three outcomes, as well as the effect of inoculation with fresh vs. stored fecal samples. By combining GC–MS metabolite profiling and 16 s rRNA gene-based amplicon sequencing, we established that starting cell densities below 10^10^ cells/ml opened up growth opportunities for members the *Enterobacteriaceae* family. This effect was exacerbated when using fecal samples that were stored frozen at −80°C. Overgrowth of *Enterobacteriaceae* resulted in lowered alpha-diversity and larger community drift, possibly confounding results obtained from fermentations in such conditions. Higher medium nutrient concentrations were identified as an additional factor contributing to inoculum community preservation, although the use of a less nutrient dense medium increased the impact of fiber product addition on the obtained metabolite profiles. Overall, our microbiome observations indicated that starting cell densities of 10^10^ cells/ml limited opportunities for exponential growth, suppressing *in vitro* community biases, whilst metabolome incubations should preferably be carried out in a diluted medium to maximize the impact of fermentable substrates.

## 1. Introduction

The human gut microbiota is a dense, complex community comprising up to 10^14^ microorganism belonging to a broad range of genera (500–1,000 across individuals) fit to thrive under the conditions of temperature, pH range, nutrient composition, and redox potential/oxygen concentration that define the colon environment (Falony et al., 2016; Poppe et al., 2021). Variation of these abiotic factors translates in changes in microbiota composition with potential impact on host health and wellbeing, opening perspectives for the design of targeted modulation strategies. Exploring these perspectives requires mechanistic insights in community dynamics (Vieira-silva et al., 2019; Vieira-Silva et al., 2020). To gain such essential insights, *in vivo* intervention trials remain the gold standard, despite having the disadvantages of being highly resource-and time consuming. Due to the high degree of inter-individual variation in microbiota composition, large numbers of volunteers are required. Moreover, avoiding more invasive sampling strategies, the effect of modulation approaches is mostly evaluated based on the fecal microbiome, representing the end-point of the colonic fermentation process and not allowing dissection of the preceding growth dynamics (Falony et al., 2018). Hence, *in vitro* fermentation strategies are considered an interesting alternative to bypass these issues. As such, a range of fermentation systems spanning from simple batch fermentations to more chemostat-like complex set-ups have been developed over the years (Venema and van den Abbeele, 2013; Vrancken et al., 2019). While more advanced simulators aim at mimicking the *in vivo* reality as accurately as possible, their complexity comes at the expense of throughput. In contrast, batch systems have a clear cost and throughput advantage, but are often more limited with respect to optimal process control in terms of pH and accumulations of metabolites. However, considering the fecal microbiota as the endpoint of a process of ecosystem maturation (rather than a sample of a continuously mixed population inhabiting the large intestine), dynamics as observed in batch systems can be hypothesized to resemble the plug-flow conditions that determine colon ecology (Falony et al., 2019).

Storage of starting material, cell density, and fermentation medium all have been described independently as factors affecting the outcome of fermentation experiments inoculated with single bacterial species as well as complex communities (Bircher et al., 2018; Atolia et al., 2020). When describing *in vitro* set-ups with fecal slurries, however, investigators rarely present the selection processes applied to identify the optimal fermentation conditions for their experiments. Such (often trial-and-error based) screening processes usually consist of a time-consuming and laborious series of small-scale incubations. Here, we present a structured way to investigate how fermentation conditions influence batch incubation dynamics. By providing insights in a process that often remains too obscure, we aim to facilitate the selection of appropriate incubation conditions for researchers planning to set up batch fermentation experiments with fecal material.

## 2. Results

To determine optimal fermentation conditions for future batch experiments with fecal material, we designed a range of explorative incubations, evaluating the effects of storage (fresh vs. stored fecal material) and concentration (bacterial cells/ml) of the inoculum on fermentation outcome. Homogenized fecal material was obtained from a single healthy donor, aliquoted, and used to start up a set of 36 fermentations. To explore the effect of dilution and frozen storage (1 week at −80°C) of the starting material on fermentation outcome, inocula encompassed fresh fecal slurry as literature-established gold standard (processed within 2 h post sampling; 1/10 w/v in physiological solution [PS; 9 mg NaCl/ml]; inoculum cell concentration of 10^11^ cells/ml), fractions obtained from a fecal dilution series of fresh feces (1/10 dilution series starting from 10^10^ to 10^7^ bacterial cells/ml), stored aliquots obtained from the same dilution series (10^8^ and 10^7^ cells/ml), and two suspensions prepared out of a 125 mg aliquot of stored fecal material (10^8^ cell/ml and a 1/10 dilution to 10^7^ cells/ml). For each fermentation, 1 ml of inoculum was added to 9 ml of medium. Per inoculum (*n* = 9), fermentations were performed in triplicate in Luria Broth (LB) supplemented with 1 g/L of pectin (citrus pectin; degree of methylation, 52.5%; galacturonic acid content, 78.1%). In addition, per inoculum, one fermentation was started up in LB without pectin as a control. Fermentations were incubated at 37°C in an anaerobic cabinet (80% N_2_, 10% CO_2_, and 10% H_2_); samples were taken 24, 48, and 96 h after inoculation and subjected to 16S rRNA gene-based amplicon sequencing to determine the optimal time-range for subsequent batch fermentation experiments.

**Figure 1:**
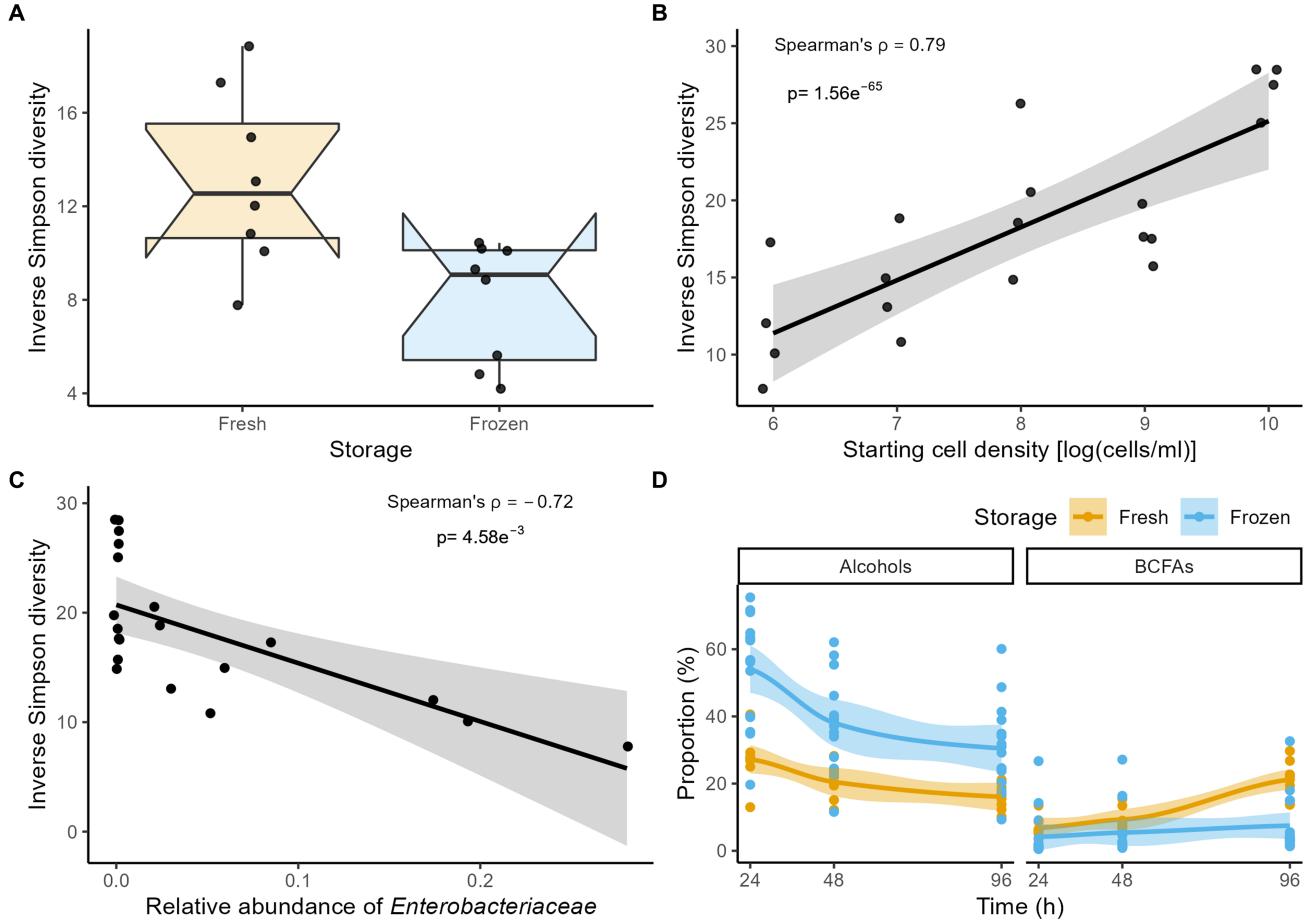
**(A)** Comparison of the Inverse Simpson diversity after 96 h of incubation between fermentations inoculated with fresh and stored material for the combined 10^7^ and 10^6^ cells/ml starting conditions (n = 16). **(B,C)** Correlation (Spearman’s ρ) between the Inverse Simpson diversity and **(B)** starting cell densities and **(C)** relative abundances of Enterobacteriaceae in fermentations inoculated with fresh material after 96 h of incubation (*n* = 20). **(D)** The time-course of the proportion (%) of alcohols and BCFAs stratified by storage condition across incubations (*n* = 108). For visualization purposes, LOESS smoothing was added.

Across fermentations and time points, the largest effect sizes in genus-level community variation were observed for differences in starting cell densities (dbRDA on the Aitchison distance matrix, *n* = 108, R^2^ = 0.13, p_adj_ = 2.0e^−6^; Supplementary Table S1), followed by the fresh/stored nature of the inocula used (R^2^ = 0.11, p_adj_ = 2.0e^−6^). When comparing the incubations of fractions obtained from the fresh and stored dilution series overlapping in terms of starting cell density (10^7^–10^6^ cells/ml), frozen storage of inocula resulted in lower community diversity in 96 h fermentation outcomes (Inverse Simpson, Mann–Whitney U test, n_96h_ = 16, r = 0.630, p_adj_ = 1.04e^−2^; Figure 1A). Similarly, focusing on the fresh dilution series encompassing the broadest range of starting cell concentrations (including fecal slurry incubations), inoculum density was observed to correlate positively with diversity after 96 h of fermentation (Inverse Simpson, n_96h_ = 20, Spearman ρ = 0.79, p_adj_ = 4.95e^−5^; Figure 1B). The observed decrease in diversity correlated with increased relative abundances of *Enterobacteriaceae* (Spearman ρ = −0.72, p_adj_ = 4.58e^−3^; Figure 1C; Supplementary Table S2), reaching highest proportions in the 10^6^ and 10^7^ cells/ml starting cell count conditions, but remaining barely detectable in the >10^8^ cells/ml incubations.

Fermentation time had a significant albeit modest impact on genus-level community variation as a whole (dbRDA, *n* = 108, R^2^ = 0.01, p_adj_ = 5.57e^−3^; Supplementary Table S1). The evolution of the relative abundances of taxa was inoculum-dependent. In fermentations inoculated with fresh material, the largest increase was observed for *Peptostreptococcus* (Friedman with pairwise Durbin-Conover test, *n* = 60, Kendall’s W = 0.72, p_adj_ = 1.6e^−5^; pairwise comparisons in Supplementary Table S3) followed by *Dialister* (Kendall’s W = 0.28, p_adj_ = 1.5e^−2^). *Clostridium* XI (Kendall’s W = 0.25, p_adj_ = 1.9e^−2^) and *Blautia* (Kendall’s W = 0.39, p_adj_ = 3.0e-3) relative abundances decreased. In incubations of stored material, *Parabacteroides* and *Clostridium* XIVa (n = 48, Kendall’s W = 0.40, p_adj_ = 9.3e^−3^ and Kendall’s W = 0.33, p_adj_ = 1.8e^−2^, respectively) increased, while proportions of unclassified *Enterobacteriaceae* decreased (Kendall’s W = 0.36, p_adj_ = 1.4e^−2^). Relative abundances of *Flavonifractor* were observed to rise in incubations of both stored and fresh material (fresh, *n* = 60, Kendall’s W = 0.44, p_adj_ = 1.3e^−3^; stored, *n* = 48, Kendall’s W = 0.78, p_adj_ = 5.0e^−5^). Community diversities increased significantly over time after inoculation with stored material (Inverse Simpson, *n* = 48, Kendall’s W = 0.77, p_adj_ = 9.57e^−6^), an association that was absent in overall more diverse incubations of freshly collected feces (*n* = 60, Kendall’s W = 0.032, p_adj_ = 0.55; Supplementary Table S2).

In contrast with the observed changes in genus-level taxa relative abundances over time, larger fluctuations in overall metabolite profiles could be observed between the 24, 48, and 96 h time points (dbRDA on Euclidian distance matrix *n* = 108, R^2^ = 0.11, p_adj_ = 1.33e^−6^; Supplementary Table S4). The most striking changes in metabolite class concentrations concerned a decrease in alcohols (Friedman with pairwise Durbin-Conover test, *n* = 108 Kendall’s W = 0.67, p_adj_ = 1.69e^−10^), combined with an increase in branched-chain fatty acids (Kendall’s W = 0.90, p_adj_ = 8.12e^−15^; Figure 1D; Supplementary Table S5). Mirroring microbiome observations, overall metabolite profiles were significantly impacted by starting cell densities and the fresh/stored nature of the inocula. Stored inocula mainly resulted in higher relative concentrations of sulfur-containing (Mann–Whitney U-test, n_96h_ = 24, r = 0.76, p_adj_ = 1.9e^−4^) and heterocyclic compounds (r = 0.68, p_adj_ = 1.85e^−3^; Supplementary Table S6), while proportions of branched-chain fatty acids (BCFAs) remained lower (r = −0.63, p_adj_ = 4.2e^−3^) when compared to fermentations inoculated with fresh material. Similarly, in incubations initiated with fresh material, proportions of short-chain fatty acids (SCFAs) and benzenoid compounds positively correlated with starting cell density (n_96h_ = 20, Spearman ρ = 0.74, p_adj_ = 6.03e^−4^ and Spearman ρ = 0.72, p_adj_ = 7.80e^−4^, respectively; Supplementary Table S6). A negative association was observed for sulfur-containing (Spearman ρ = −0.83, p_adj_ = 4.93e-5) and heterocyclic compounds (Spearman ρ = −0.80, p_adj_ = 1.01e^−4^).

The initial, explorative analyses described above indicated an overgrowth of the family *Enterobacteriaceae* and lower community diversity in fermentations inoculated with stored material and with lower inoculum cell counts. Fermentation time only had a limited impact on microbiome variation within the 24–96 h sampling window. Based on these observations, we designed a single-donor full factorial follow-up experiment, refocusing the sampling window and increasing frequency to 0, 6, 12, and 24 h. Variation in initial cell densities in the fermentation medium was narrowed to 10^8^–10^10^ cells/ml. Besides LB, we added a ½ diluted medium as well as a physiological saline (PS) arm to the design to evaluate the effect of nutrient density on the microbiota and metabolite profiles. Fermentations were inoculated with fresh or stored inocula, with or without supplementation of the medium with 1 g/L of pectin. Incubations were carried out in duplicate, resulting in a total of 72 experiments.

**Figure 2:**
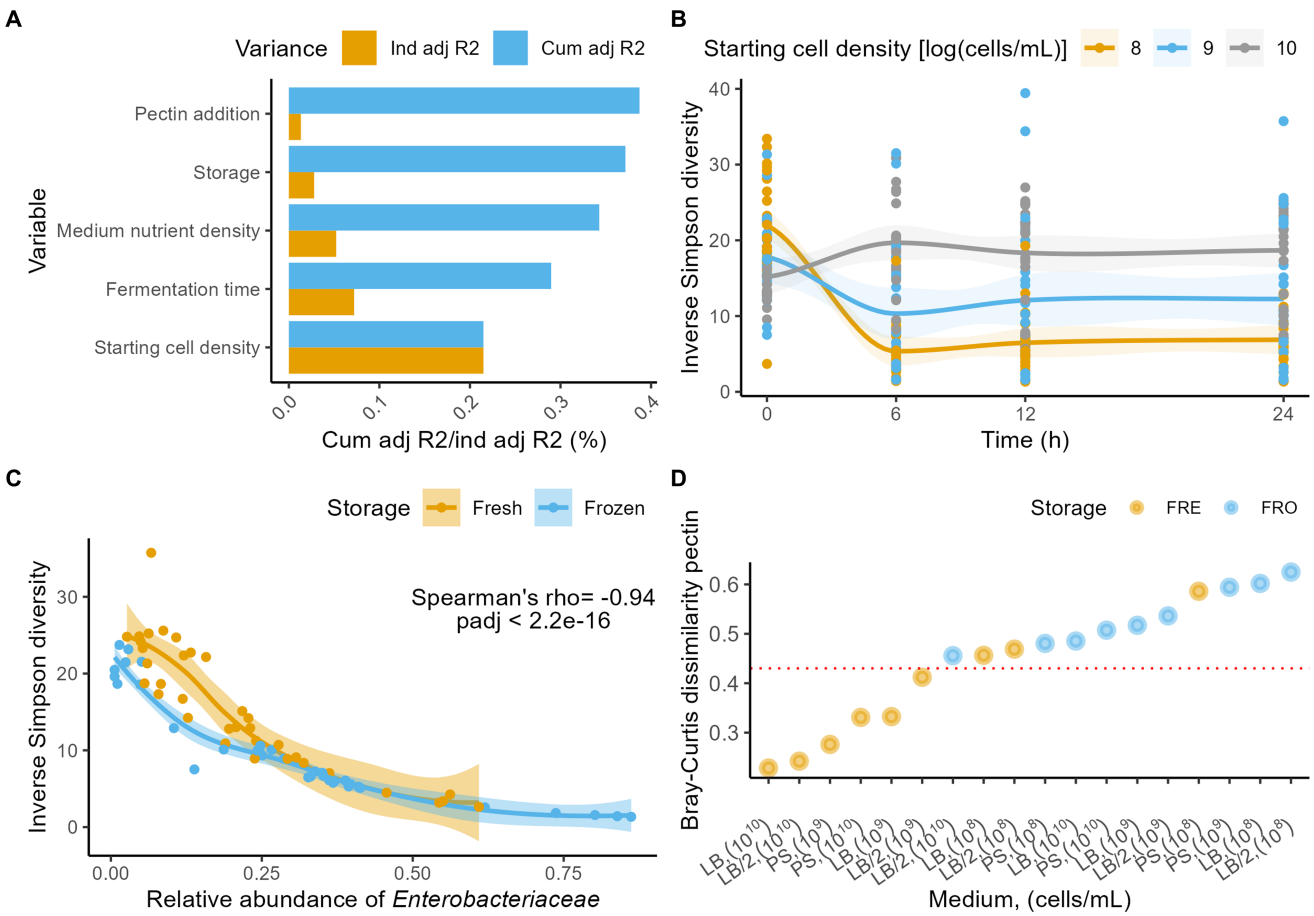
**(A)** Independent (yellow) and cumulative, non-redundant (blue) effect sizes (adjusted R2) of time and fermentation conditions on genus-level microbiome community variation (dbRDA on the Aitichon distance matrix, *n* = 288). **(B)** Evolution of the Inverse Simpson diversity over 24 h stratified by starting cell densities (*n* = 288). LOESS smoothing was applied for visualization. **(C)** Correlation (Spearman’s ρ) between the relative abundances of Enterobacteriaceae and the Inverse Simpson diversity at 24 h (*n* = 72), stratified by storage conditions. LOESS smoothing was applied for visualization. **(D)** Bray-Curtis dissimilarities between the 0 and 24 h time point stratified per condition (starting cell density, medium, storage) for the fermentations without pectin (*n* = 36). The horizontal red line indicates the 0.45 **(B,C)** dissimilarity threshold above which only incubations started with stored material or with initial cell densities of <10^8^ cells/ml were scored.

As expected, given the narrowed, more focused set-up of the full-factorial experiment, the explanatory power of fermentation conditions on microbiome variation increased compared to the exploratory trial. Fermentation time was observed to have the second largest effect size in microbiome variation (dbRDA on Aitchison distances, *n* = 288, R^2^ = 0.08, p_adj_ = 5.0e^−6^), topped by starting cell count (R^2^ = 0.22, p_adj_ = 5.0e^−6^) and followed by medium nutrient density (i.e., LB, LB_/2_, or PS; R^2^ = 0.06, p_adj_ = 1.2e^−4^), fresh/stored nature of the inoculum (R^2^ = 0.03, p_adj_ = 1.74e^−3^; Figure 2A), and addition of pectin (R^2^ = 0.02, p_adj_ = 2.2e^−2^; Supplementary Table S7). Across fermentations, the community diversity decreased in function of fermentation time (Inverse Simpson, Friedman with pairwise Durbin-Conover test, *n* = 288, Kendall’s W = 0.12, p_adj_ = 1.07e^−5^; Supplementary Table S8). This decrease was however mostly driven by microbiome variation as observed in the 10^8^ and 10^9^ cells/ml starting cell count conditions, since no significant association between diversity and time could be established in the 10^10^ cells/ml fermentations (10^8^ cells/ml, Friedman with pairwise Durbin-Conover test, *n* = 96, Kendall’s W = 0.57, p_adj_ = 2.0e^−8^; 10^9^ cells/ml, *n* = 96, Kendall’s W = 0.35, p_adj_ = 1.8e^−5^; 10^10^ cells/ml, n = 96, Kendall’s W = 0.107, p_adj_ = 0.052; Figure 2B; Supplementary Table S8). Excluding the 10^10^ cells/ml condition, we observed a decrease in diversity over time for both fresh and frozen material (fresh, Friedman with pairwise Durbin-Conover test, *n* = 96, Kendall’s W = 0.24, p_adj_ = 5.99 e^−4^; stored, *n* = 96, Kendall’s W = 0.72, p_adj_ = 6.14e^−11^; Supplementary Table S8). Overall, the diversity observed after 24 h of incubation appeared to be tightly linked to *Enterobacteriaceae* relative abundances (n_24h_ = 72, Spearman ρ = −0.94, p_adj_ = 2.2e^−16^; Figure 2C; Supplementary Table S9).

While the evolution of community diversity allows to screen for major macro-ecological perturbations such as population overgrowth, it does not provide insights in the dynamics of individual taxa. One could even envisage a scenario where a microbial population maintains a constant level of diversity, but where all taxa present in the ecosystem get replaced by opportunistic colonizers. To quantify drift from starting profiles, we evaluated the Bray–Curtis dissimilarity distances between time points 0 and 24 h of all incubations. For all fermentations inoculated with stored material and for those with a starting cell density of 10^8^ cells/ml, dissimilarity was higher than 0.45, indicating a substantial drift away from the initial microbiota composition (Figure 2D; Supplementary Table S10). Lowest dissimilarities (<0.30) were observed for starting densities of 10^10^ cells/ml, incubated in either LB or LB_/2_.

**Figure 3:**
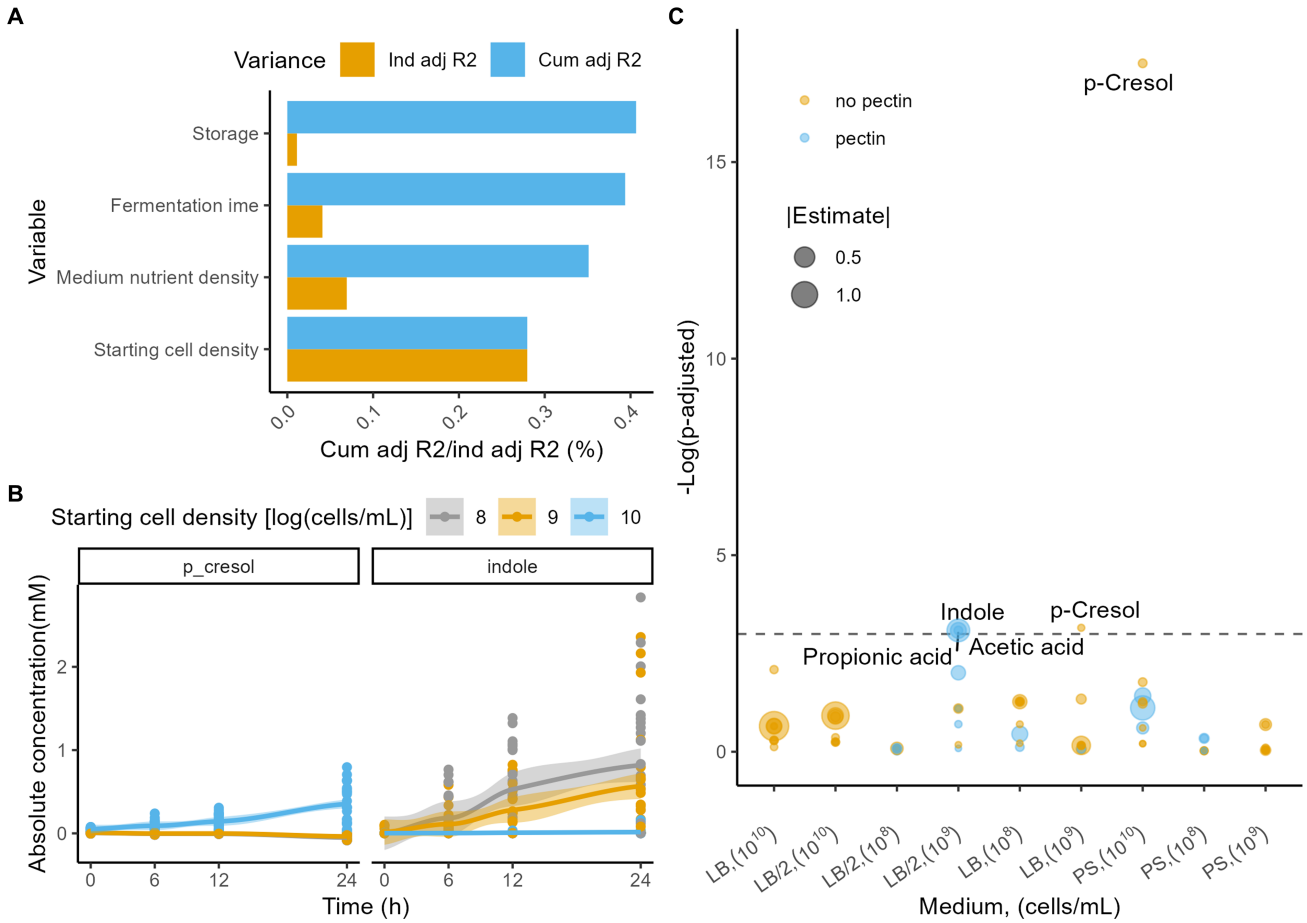
**(A)** Independent (yellow) and cumulative, non-redundant (blue) effect sizes (adjusted R2) of time and fermentation conditions on metabolome profile variation (dbRDA on the Euclidian distance matrix, *n* = 288). **(B)** Evolution of the absolute concentrations (mM) of the metabolites indole and p-cresol stratified per starting cell densities [log(cells/ml)]. LOESS smoothing was applied for visualization purposes. **(C)** Linear model estimates of the differences in metabolite production rates (mM/h) across fermentations (all time points), with and without addition of pectin, stratified per medium and starting cell density (*n* = 288). Colors indicate whether the rate was higher with pectin, whilst sizes correlate with the estimate. The horizontal dashed line indicates the p_adj_ = 0.05.

Fermentation metabolite profiles were most strongly impacted by starting cell density (dbRDA on Euclidian distance matrix, *n* = 288, R^2^ = 0.28, p_adj_ = 2.5e^−6^;), followed by medium nutrient density (R^2^ = 0.076, p_adj_ = 2.5e^−6^) and fermentation time (R^2^ = 0.044, p_adj_ = 2.5e^−6^; Figure 3A; Supplementary Table S11). In contrast with microbiome observations, pectin addition did not have an effect on metabolite profiles (R^2^ = 0.002, p_adj_ = 0.576), and the effect size of inoculum storage was smaller (R^2^ = 0.01, p_adj_ = 0.016). Concentrations of all absolutely quantified metabolites increased over time, with the largest effect sizes observed for butyric acid (Friedman test, *n* = 288, Kendall’s W = 0.742, p_adj_ = 1.7e^−33^), iso-valeric acid (Kendall’s W = 0.566, p_adj_ = 1.3e^−25^), and indole (Kendall’s W = 0.464, p_adj_ = 1.3e^−25^; Supplementary Table S12). Meanwhile, the putative deleterious protein fermentation metabolite para-cresol only showed limited variation over time (Kendall’s W = 0.0566, p_adj_ = 6.6e^−3^). Interestingly, para-cresol increased only in the 10^10^ cells/ml condition (Friedman with pairwise Durbin-Conover test, *n* = 96, Kendall’s W = 0.736, p_adj_ = 5.5e^−11^), while decreasing in the 10^8^ and 10^9^ cells/ml incubations (10^8^ cells/ml, *n* = 96, Kendall’s W = 0.7, p_adj_ = 3.3e^−7^; 10^9^ cells/ml, *n* = 96, Kendall’s W = 0.457, p_adj_ = 9.8e^−11^; Figure 3B; Supplementary Table S12), resulting in a significant difference at the end of the fermentation (Kruskal-Wallis with *post hoc* Dunn-test, n_24h_ = 72, eta^2^[H] = 0.69, p_adj_ = 3.0e^−4^; Supplementary Table S13). In contrast, indole increased in all conditions, but was significantly higher after 24 h of fermentation for the 10^8^ and 10^9^ cells/ml starting cell count conditions (n_24h_ = 72, eta^2^[H] = 0.21, p_adj_ = 8.8e^−11^; Supplementary Table S13). The discrepancy between indole and para-cresol observations in the 10^10^ and the 10^8^–10^9^ cells/ml starting cell count conditions might, along with the difference in diversity dynamics and *Enterobacteriaceae* bloom, point to a difference in growth stage for the microbiota.

To evaluate the effect of pectin addition on the absolute concentrations of metabolites through time of fermentation, we modelled the experiments stratified per medium and starting cell density (linear modelling [LM]). Pectin addition had a significant effect on at least one of the metabolites’ concentration in 3 of the 9 combinations of conditions evaluated (Figure 3C). For the LB_/2_ medium with a starting cell density of 10^9^ cells/ml, pectin addition significantly increased the concentrations of acetic acid (LM, *n* = 32, estimate = 0.695 mM/h, p_adj_ = 0.045), propionic acid (estimate = 0.230 mM/h, p_adj_ = 0.045), and indole (estimate = 0.0143 mM/h, p_adj_ = 0.045) produced during the fermentation.

Overall, based on microbiome observations, the results of the full factorial experiment indicate that in order to preserve the fecal microbiota composition over the course of batch fermentation experiments, the use of a freshly prepared inoculum, incubated in LB or LB_/2_ at a starting cell count of 10^10^ cells/ml is recommended. Metabolome findings indicate that, in order to follow up the effect of the addition of a fermentable substrate (i.e., pectin), incubations should preferably be carried out in a diluted medium. To further validate the workflow proposed for the key endpoint of community representativeness - tightly coupled to controlling opportunistic colonizer proportions – we performed 12 additional fermentations inoculated with fecal material from different donors spanning all four commonly detected enterotypes (Vandeputte et al., 2017). Carried out in LB_/2_ medium without pectin with a starting cell density of 10^10^ cells/ml, proportions of *Escherichia* were determined after 24 h of fermentation using 16S rRNA gene-based amplicon sequencing. Resulting proportions of *Escherichia* varied between 0 and 19% (median abundance of 3%) – comparable to the 6% in the corresponding condition of the full factorial experiment. This demonstrates that our workflow is generally applicable across community types, taking into account the variability of individual donors (Supplementary Table S15).

## 3. Discussion

Assessing the impact of fermentation conditions on the outcome of incubations of fecal material, we observed starting cell density to be the major driver of end-point community as well as metabolite profile variation, followed by storage of inocula and medium nutrient density. A key factor for the interpretation of our findings is presumably the carrying capacity of the fermentation set-up, i.e., the maximum population density an environment can support (Nowak, 2006). Inoculation of the fermentation medium to a starting density of 10^10^ cells/ml appeared to approximate or even exceed the maximal load supported by the batch systems analyzed. On one hand, such a set-up confined the growth of opportunistic colonizers. On the other hand, while high-density incubations conserved the initial composition of the inoculum, they also limited exploration of the dynamics of ecosystem development. Lower starting cell densities (<10^10^ cells/ml) did provide opportunities for exponential growth of inoculated species, but the outcome of these fermentation experiments was characterized by lower microbial diversities and high proportions of *Enterobacteriaceae*. Representing a phylogenetic family of opportunistic pathogens, the latter mostly display short lag times and high growth rates (Pompan-Lotan et al., 2015; Milani et al., 2017). By lowering starting cell densities below the carrying capacity of fermentation set-up, an opportunity was created for such opportunistic colonizers to dominate the bacterial community (Monod, 1949; Jabot and Pottier, 2012) This effect was potentially exacerbated by the short but substantial oxygen and temperature shock the microbiota was exposed to after egestion and during inoculum preparation. Freezing the inoculum before fermentation compounded this stress, adding longer lag times to the equation (Atolia et al., 2020). This further benefited *Enterobacteriaceae* (and potentially other opportunists), pushing the endpoint community profile further from being representative for fecal ecosystem. Of note: while freezing stress mostly results from freeze/thaw cycles (leading to crystal formation and development of osmotic gradients), residual traces of unfrozen water at storage temperatures above −135°C could induce additional biological variation tightly linked to duration of frozen storage (Hubálek, 2003; Heylen et al., 2012). However, the latter was not investigated in the scope of the present study.

From an ecosystem development perspective, starting cell densities below or at system carrying capacity represent two distinct stages of fecal community development (Falony et al., 2018). Lower densities correspond with an immature configuration, where the relative fitness of individual taxa mostly depends of their ability to outcompete others in terms of nutrient consumption as well as growth rates (Costello et al., 2012). The associated ecosystem dynamics not only occur during initial colonic colonization of the chime matrix, but also determine the composition of the fecal microbiota of individuals with short transit times. *In vitro*, where the outcome of inter-taxon competition is not subjected to host control mechanisms such as immune surveillance, the end result of the community maturation process initiated by inoculum dilution unfortunately differed substantially from what has been observed *in vivo*, due to the unhampered overgrowth of opportunistic colonizers (Ho et al., 2017; Byndloss et al., 2018). Hence, the most effective way to generate an *in vitro* equivalent of a mature microbiota is to incubate (preferably freshly harvested) fecal material at cell concentrations approaching the maximal load supported by the set-up envisaged.

Regarding metabolite profiles, also the observed trade-off between indole and para-cresol production might reflect the difference in ecological development stage hypothesized to result from high-versus low-density inoculations. Indole production, mostly observed in our incubations with a starting cell density < 10^10^ cells/ml, has been associated with fast-growing *Enterobacteriaceae* (Lee and Lee, 2010). Para-cresol, observed in high-density inoculations potentially corresponding with more mature ecosystem configurations, has been traced back to more slow-growing clostridial clusters (Saito et al., 2018). Similarly, alcohol production has been linked to fast-growing saccharolytic species (Boumba et al., 2008; Elshaghabee et al., 2016) while branched-chain fatty acids are considered markers of protein fermentation (Macfarlane et al., 2006)– again aligning the observed proportional changes in metabolite production between low-and high-density inoculations with the expected dynamics of ecosystem maturation (Falony et al., 2018). Proportional depletion of the more readily degradable carbohydrate substrates with respect to the amount of amino acids and peptides present in the fermentation medium would indeed push the microbial community towards the energetically less favorable protein fermentation. The ratio of carbohydrates to amino acids and peptides in the medium also provides an explanation to the more pronounced effect of pectin addition in a more dilute medium. At a constant concentration of the added carbohydrate substrate (pectin), an increased concentration of peptides and amino acids lowers the Gibbs free energy of protein relative to that of carbohydrate fermentation, hence lowering the energetic benefit of carbohydrate fermentation.

## 4. Conclusion

Here, we optimized the fermentation conditions for *in vitro* fermentations with fecal inocula by systematically investigating the effect of starting cell density, medium nutrient density, and fresh inoculation versus inoculation after frozen storage on the metabolite and microbiota profiles. The aim of the optimization – here in the context of fermentations with fiber products - was to minimize drift from the starting inoculum in the condition without fiber product and to maximize the impact of the addition of fiber product on the fermentation characteristics. The optimization revealed that starting cell densities of 10^10^ cells/ml maximally lowered *in vitro* drift, but possibly limited exponential growth of taxa. Meanwhile, the addition of a more dilute medium (LB_/2_) maximized the impact of fiber product addition on the metabolite profiles. Above all, we have provided a framework for other researchers to set up their own fermentations systems, and optimize for their specific set-up and research question.

## 5. Materials and methods

### 5.1. Fermentation method

We performed the fermentation experiments anaerobically in a Don Whitley A35 Anaerobic Workstation with HEPA filter (Don Whitley Scientific) with a 80% N_2_, 10% CO_2_, and 10% H_2_ atmosphere at a pressure of 3 bar and a temperature of 37°C, in autoclave sterilized Hungate anaerobic tubes (Chemglass Life Sciences). The medium was incubated in the anaerobic chamber for over 24 h to dissipate residual oxygen.

### 5.2. Preparation of medium

All media were pH controlled to pH 6 and a 1.25 g/L concentration of sodium bicarbonate (Thermo Fisher Scientific) as the buffer. One medium consisted of physiological water, a second one consisted of 20 g/L of Luria Broth (LB; Thermo Fisher Scientific) and a final one of 10 g of LB per liter, with an added 2.5 g of NaCl (Merck) as an osmotic agent. LB medium without added carbohydrates was used as a reference fermentation, whilst to the other media 1 g/L of citrus pectin (degree of methylation: 52.5%, galacturonic acid content: 78.1%; Cargill) was added.

### 5.3. Fecal samples and preparation of inocula

Fecal samples were obtained from a single healthy donor (not receiving antibiotic treatment for at least 3 months, not consuming pre-or probiotic containing supplements prior to experimentation, and without a history of intestinal disorders) and processed within 2 h after collection. Fractions of the fresh samples were homogenized (10% w/V) in PS (9 mg NaCl/ml; Baxter), 20 g/L Luria broth (LB) and 10 g/L (LB½) in the anaerobic workstation. The fecal slurry dilution series were obtained by adding 1 ml of the fecal slurry to 9 ml of the relevant medium, continuing from 10^11^ to 10^7^ cells/ml. Aliquots of each fecal slurry or fecal slurry dilution, as well as 125 mg aliquots of fecal material were immediately frozen at −80°C for 1 week. Fresh fermentations were inoculated within 30 min of the preparation of the dilution series, whilst the frozen fermentations were started after thawing in the anaerobic workstation.

### 5.4. GC – MS method

Metabolites were analyzed using a headspace – cold trap – gas chromatography system coupled to a mass detector. Immediately before analysis, the fermentation samples were thawed and homogenized. 1 ml of the fermentation sample was transferred into GC headspace vials. As an internal standard, 50 μl of 2 ethyl-butyrate (125 mg/100 ml; Merck), diethyl sulfide (25 mg/100 ml; Merck), and 2,6 dimethyl phenol (12,5 mg/100 ml; Merck) were added. To salt out and acidify the solution, 300 mg of sodium chloride and 125 μl of 98% sulphuric acid (Merck) were added, respectively. GC vials were heated to 60°C for 5 min using a Triplus RH auto sampler (Thermo Fischer Scientific) after which 1 ml of the headspace was injected in a Trace GC ultra gas chromatograph equipped with a cold-trap system. For separation, a RT wax (cross-bonded carbowax PEG; Restek) column with 0,25 μm df, 0,25 mm ID and a length of 30 meter was used. The separated compounds were then detected using a DSQ II electron impact, single quadrupole mass spectrometer. Select compounds were absolutely quantified using external standards. Data collection and absolute quantification of the selected compounds was performed using Xcalibur software (Thermo Fisher Scientific), whilst metabolite identification was performed using Analyzer pro software (Spectral Works). Compounds were identified by comparing their mass spectra to the NIST library, with manual validation based on expert knowledge. For further analysis, components were binned into chemical classes. Subsequently, relative indices were calculated compared to the 2-ethyl butyrate internal standard. This method was adjusted for the head space-cold-trap system from De Preter et al. (2009).

### 5.5. Microbiota sequencing

16 s rRNA sequencing was performed as described by Tito et al. (2017). Total bacterial DNA was extracted from the fermentation samples using a MagAttract PowerMicrobiome DNA/RNA KF kit (QIAGEN) following the manufacturer’s instruction. All steps were performed under a biohazard type II cabinet, and all material was decontaminated using ultraviolet C light. Surfaces and gloves were decontaminated using RNase AWAY (Molecular Bio-Products). To amplify the V4 region of the 16S rRNA gene, we used 515F and 806R primers (GTGYCAGCMGCCGCGGTAA and GGACTAC-NVGGGTWTCTAAT, respectively), modified to contain Illumina adapters and dual barcode sequences to allow for directional sequencing (Apprill et al., 2015). Sequencing was performed at the VIB Nucleomics Core laboratory (Leuven, Belgium) with a HiSeq 2500 system (151 bp paired-end reads) and Illumina MiSeq platform (MiSeq Reagent Kit v2) for the exploratory and full factorial experiment, respectively in accordance with the manufacturer’s specifications, to generate paired-end reads in each direction. Overlapping paired-end reads were merged and processed with the LotuS pipeline (Hildebrand et al., 2014). DADA2 was used to merge paired sequences and remove chimeras before grouping them into Amplicons Sequence Variants (ASVs; Callahan et al., 2016). The RDP classifier (version 16) was used for taxonomic assignment (Wang et al., 2007).

### 5.6. Flow cytometry

For cell counting, 200 μl of fermentation suspension was diluted 100.000 times in PS. In the case of too few events per μl, a 10 fold lower dilution was utilized. One ml of the microbial cell suspension obtained was stained with 1 μl SYBR Green I (1:100 dilution in dimethylsulfoxide; shaded 20 min incubation at 37°C; 10.000 concentrate; Thermo Fisher Scientific). The flowcytometry analysis of the microbial cells present in the suspension was performed using a C6 Accuriflow cytometer (BD Biosciences), according to previously published methods (Vandeputte et al., 2017). Fluorescence events were monitored using the FL1 533/30 nm and FL3 > 670 nm optical detectors. Forward and sideways-scattered light was also collected. The BD Accuri CFlow software was used to gate and separate the microbial fluorescence events on the FL1–FL3 density plot from the sample background. A threshold value of 2000 was applied on the FL1 channel. The gated fluorescence events were evaluated on the forward–sideways density plot, to exclude remaining background events and to obtain an accurate microbial cell count. Instrument and gating settings were identical for all samples.

### 5.7. Statistical analysis

All statistical analyses were performed using R version 4.1.3. α-Diversity (inverse Simpson) was calculated using the vegan package (Oksanen et al., 2019). Microbiota and metabolite features were removed if they were absent in >80% of the samples, whilst zero values were imputed with half of the minimum recorded feature value. To estimate non-redundant variation explained by fermentative conditions, stepwise distance-based redundancy analysis (dbRDA, vegan package) was used, determining *p*-values with 1,000 permutations. For the microbiota, this was performed on the genus-level Aitchison distance matrix, after clr-transformation, whilst for metabolites it was performed on the Euclidian distance matrix. For comparisons between multiple groups, Kruskall-Wallis with post-hoc Dunn tests were used, otherwise the Mann Whitney-U test was used. For within group comparisons Friedman tests with post-hoc Durbin-Connover tests were used using the PMCMRplus package (Pohlert, 2022). Correlations between continuous measures were assessed using Spearman’s rank order correlation. All *p* values were corrected for multiple testing when appropriate using the Benjamini–Hochberg method (p_adj_), only p_adj_ < 0.05 were reported as significant. Visualisations were enhanced using ggplot2 (Wickham, 2016). The Rstatix package was used to optimize basic statistical testing across multiple variables (Kassambara, 2021).

## Data availability statement

The datasets presented in this study can be found in online repositories. The names of the repository/repositories and accession number(s) can be found at: https://www.ebi.ac.uk/ena, PRJEB58105.

## Ethics statement

The studies involving human participants were reviewed and approved by Ethics Committee Research UZ/KU Leuven (reference number S65767). The patients/participants provided their written informed consent to participate in this study.

## Author contributions

JP, GF, and KV performed the conception and design of the study. JP performed the data collection and experimental work. JP and SV-S performed the data preparation. JP, SV-S, and GF performed the statistical and data analysis. JP, SV-S, JR, and GF performed the interpretation. JP, SV-S, GF, and KV drafted the manuscript. All authors contributed to the article and approved the submitted version.

## Funding

JP is funded by a doctoral fellowship from Flanders Innovation & Entrepreneurship (VLAIO) (HBC.2017.0596), while GF is funded by the ReALity Innovation Fund, Research Initiative of the State of Rhineland-Palatinate, Germany.

## Conflict of interest

JR is an advisor for MRM health.

The remaining authors declare that the research was conducted in the absence of any commercial or financial relationships that could be construed as a potential conflict of interest.

## Publisher’s note

All claims expressed in this article are solely those of the authors and do not necessarily represent those of their affiliated organizations, or those of the publisher, the editors and the reviewers. Any product that may be evaluated in this article, or claim that may be made by its manufacturer, is not guaranteed or endorsed by the publisher.
